# Low-level proteinuria at the initial diagnosis of systemic lupus erythematosus significantly increases the risk of developing proteinuria-predominant lupus nephritis

**DOI:** 10.1371/journal.pone.0361288

**Published:** 2026-10-29

**Authors:** Minghui Song, Guolong Xu, Fengxian Xu, Shengkai Zhang, Qinqin Wang, Wenjing Xue, Ruiying Chen, Lixin Wei, He-Jun Li

**Affiliations:** 1 Department of Rheumatology, Sanming First Hospital Affiliated to Fujian Medical University, Sanming, China; 2 Department of Rheumatology, Fujian Medical University Union Hospital, Fuzhou, China; 3 Department of Rheumatology, Nanping First Hospital Affiliated to Fujian Medical University, Nanping, China; 4 Department of Nephrology, Fujian Medical University Union Hospital, Fuzhou, China; 5 Fujian Institute of Clinical Immunology, Fujian Medical University Union Hospital, Fuzhou, China; Prime Hospital LLC, UNITED ARAB EMIRATES

## Abstract

**Purpose:**

A proportion of patients with systemic lupus erythematosus (SLE) present with subclinical low-level proteinuria at initial diagnosis that does not meet the formal classification criteria for lupus nephritis (LN). This study aimed to investigate the association between low-level proteinuria at SLE onset and the risk of developing proteinuria-predominant LN.

**Patients and methods:**

This was a retrospective cohort study using medical records of patients newly diagnosed with SLE from Fujian Medical University Union Hospital between December and March. Inclusion required urine dipstick proteinuria ≤2+; if quantified, protein excretion <500 mg/24h (by the urine protein-to-creatinine ratio [UPCR] or 24-hour proteinuria). Demographic, clinical, and laboratory data were collected at the time of initial diagnosis, as well as urinalysis, urine sediment, UPCR, and 24-hour proteinuria during follow-up, where available. The association between various baseline variables and the subsequent development of proteinuria-predominant LN was analyzed using a Cox proportional hazards model.

**Results:**

Of 330 patients screened, 297 were eligible for inclusion in the final analysis, of whom 85 patients had low-level proteinuria at the initial diagnosis. According to multivariable-adjusted models, low-level proteinuria significantly increased the risk of proteinuria-predominant LN (*P* < 0.001, hazard ratio[HR] = 3.355, 95% confidence interval [CI]: 1.6646.765), as did male sex (*P* = 0.001, HR = 3.178, 95% CI: 1.563–6.460).

**Conclusion:**

This study highlights that SLE patients with low-level proteinuria at initial diagnosis may require enhanced monitoring for LN, enabling early detection. Future studies are needed to determine the necessity of early intervention.

## Introduction

Systemic lupus erythematosus (SLE) is a chronic autoimmune inflammatory disease of the connective tissue,which leads to organ damage and low quality of life. The type and severity of organ involvement are critical factors in the selection of pharmacological interventions for SLE [1]. Lupus nephritis (LN), characterized by heterogeneous clinical and histopathological findings, represents one of the most severe complications of SLE and is associated with a high risk of disability and mortality [2]. Abnormal proteinuria and urinary sediment are characteristic clinical manifestations of LN. Currently, the management of lupus nephritis continues to pose a significant challenge, with treatment response and long-term prognosis remaining unsatisfactory [3]. Moreover, the kidney response to treatment in patients with a baseline urine protein-to-creatinine ratio (UPCR) of 3 g/g or more was significantly weaker compared to those with a baseline UPCR under 3 g/g [4]. Therefore, under the current treatment methods, early detection and timely intervention may be critical for improving the prognosis of the kidneys. Although kidney biopsy is the diagnostic gold standard for LN and crucial for guiding treatment, the KDIGO 2024 Guideline recommends it only for patients with 24-hour proteinuria ≥500 mg/d to account for physiological causes of low-level proteinuria and avoid unnecessary procedures [5]. Whether low-level proteinuria at baseline in newly diagnosed SLE patients represents a benign finding or carries prognostic value for the future development of proteinuria-predominant LN remains unexplored.

Consequently, this study is designed to evaluate the association between low-level proteinuria at SLE onset and the risk of developing proteinuria-predominant LN, aiming to facilitate early risk stratification and intervention.

## Methods

### Study cohort and patients

An inception cohort consisting exclusively of patients with newly diagnosed SLE was recruited from **patients** hospitalized at Fujian Medical University Union Hospital between December 2012 and March 2021. The eligibility criteria were as follows: (1) met the 2012 Systemic Lupus International Collaborating Clinics (SLICC) Classification Criteria [6];(2) were newly diagnosed with SLE during the index hospitalization; (3) received follow-up visits at the Fujian Medical University Union Hospital after discharge;(4) UPCR or 24h urine protein representing < 500 mg of protein/24h;(5) proteinuria assessed by dipstick protein ≤2 + . The exclusion criteria were as follows: (1) abnormal urine tests during hospitalization attributable to infections, bleeding, or contamination; (2) dipstick proteinuria = 2+ at inclusion screening, in the absence of confirmatory UPCR or 24-hour proteinuria measurement; (3) two consecutive findings of dipstick proteinuria = 2+ during follow-up, without confirmatory quantitative measurement (by UPCR or 24-hour proteinuria);(4) dipstick proteinuria ≥ 3+ at any point during follow-up, without confirmatory quantitative measurement (by UPCR or 24-hour proteinuria).

### Declaration

Ethics approval and consent to participate. The institutional review boards (the ethics board of Fujian Medical University Union Hospital under the 2021KY139 project number) approved all protocols. As this study was retrospective in nature, the requirement for informed consent was waived and general confidentiality principles were abided. This study was performed without direct patient and public involvement in the design phase. Access to private data can be granted upon request.

### Definitions

The primary endpoint was significant proteinuria, defined as either UPCR or 24h urine protein representing ≥ 500 mg of protein/24h, in accordance with the 2012 SLICC classification criteria for proteinuria-dominant lupus nephritis [6]. Low-level proteinuria was defined by any of the following criteria during hospital stay: (1) a negative urine dipstick result with a quantitative test (either UPCR or 24-hour urine protein) indicating a level between the upper limit of normal (UPCR 200 mg/g; 24-hour protein 120 mg) and 500 mg/g or 500 mg/24h, respectively; (2) a urine dipstick result of ‘1 + ’ and, when available, a quantitative test result of UPCR < 500 mg/g or 24-hour proteinuria < 500 mg/d; (3) a urine dipstick result of “2 + ” with the requirement of at least one subsequent quantitative test meeting the criterion of UPCR <500 mg/g or 24-hour proteinuria <500 mg/d. Based on the level of urine protein at screening, patients were divided into two groups: the negative-proteinuria group and the low-level proteinuria group.

Hematuria and pyuria were defined as the presence of >5 RBCs/HPF and >5 WBCs/HPF, respectively, in the absence of infection. Hypocomplementemia was defined as C3 or C4 below the laboratory reference range. A positive anti-dsDNA result was determined as a level exceeding the reference range established by the laboratory, in accordance with the SLICC classification criteria. A medium-to-high titer of anticardiolipin antibody (aCL) was defined as a level exceeding twice the upper limit of normal.

In this inception cohort, disease duration was defined as the time from symptom onset to the point of being newly diagnosed with SLE at our center. The follow-up period began at the first date of initial SLE diagnosis thereafter and extended until the occurrence of significant proteinuria, or the last follow-up visit, whichever occurred first. Time to significant proteinuria was defined, specifically for patients who developed significant proteinuria, as the duration from the date of initial SLE diagnosis to the date of significant proteinuria occurrence.

### Data collection

The data for this retrospective study were accessed on 01/06/2025.We obtained demographic, clinical and laboratory parameters at the time of the first diagnosis by means of a review of electronic medical records, including gender, age, disease duration, SLEDAI-2K, serum complement, anti-dsDNA, and anti-phospholipid antibody. In case of any discrepancy or missing data, confirmation was obtained via telephone follow-up. SLE-related organ involvements at the time of the first diagnosis, such as cutaneous, arthritis, serositis, lupus nephritis (LN), neuropsychiatric SLE (NPSLE), hematological involvement (leukopenia, thrombocytopenia, and hemolytic anemia), were collected from the medical records of all recruited patients. Data, including urinalysis, urine sediment, UPCR, and 24-hour proteinuria, were collected at the initial diagnosis and throughout the follow-up period, where available.

### Statistical analysis

Categorical variables were described as numbers (percentages) and compared using the Chi-square test or Fisher's exact test between the negative-proteinuria group and the low-level proteinuria group. Normally distributed continuous data were presented as the mean and standard deviation (SD), and differences between groups were tested by one-way analysis of variance. Abnormally distributed quantitative variables were expressed as medians (interquartile range [IQR]) and compared between groups using the Mann‒Whitney nonparametric U test.

The associations between the risk of significant proteinuria and factors with significant differences (*P* < 0.05) in bivariate analysis were analyzed by a univariate Cox regression model. Variables with *P*  <  0.05 in univariate Cox analysis were included in the multivariate model to adjust for potential confounders. A sensitivity analysis was conducted to address potential bias from insufficient observation time. Specifically, we excluded patients who did not reach the endpoint and had follow-up durations less than the 25th percentile (20.5 months) of the time-to-significant proteinuria distribution among those who did. All reported *P* values were two-sided, with the threshold for statistical significance set at *P* < 0.05. All statistical analyses were performed using R version 4.3.2.

## Results

### Study population

Of 330 patients screened, a total of 297 cases were included in the final analysis (Fig 1). As shown in Table 1, of the 297 patients, 85 patients had low-level proteinuria at the initial diagnosis. A total of 42 patients (14.1%) developed significant proteinuria during the study period, with 16 cases in the negative-proteinuria group and 26 in the low-level proteinuria group. Among the 42 patients who developed significant proteinuria, kidney biopsy was performed in 4 patients (1 in the negative-proteinuria group and 3 in the low-level proteinuria group). All 4 patients were classified as Class IV + V lupus nephritis according to the ISN/RPS classification. The median follow-up period was 85.5 months (IQR 57–108.75) and 70 months (IQR 28.5–103) in the negative-proteinuria group and the low-level proteinuria group, respectively. Among patients who developed significant proteinuria, 24-hour proteinuria was 1455 mg/d (IQR 742.75–5995) and 1610.5 mg/d (IQR 1004.75–4188) in the negative-proteinuria group and the low-level proteinuria group, respectively.The low-level proteinuria group had a significantly higher proportion of patients who progressed to significant proteinuria than the negative-proteinuria group (30.6% vs. 7.5%, *P* < 0.001). The median time to significant proteinuria showed a trend toward being shorter in the low-level proteinuria group compared to the negative-proteinuria group (months, IQR, 37[16.5–69.25] vs. 52.5[42.25–103], *P* = 0.054).

**Table 1:** Comparison of clinical characteristics between the negative-proteinuria group and the low-level proteinuria group by bivariate analysis.

	All(n = 297)	negative-proteinuria group(n=212)	low-level proteinuria group(n=85)	*P* value
Gender, F (%)	264(88.9)	195(92)	69(81.2)	0.007
Age at SLE diagnosis, years, median [IQR]	32[22–44]	33[22–45]	30[20.5–42]	0.128
Disease duration, Months, median [IQR]	3[1–13]	3[1–13]	2[1–7]	0.115
Fever, n(%)	95(32)	59(27.8)	36(42.4)	0.015
Cutaneous, n(%)	142(47.8)	94(44.3)	48(56.5)	0.059
Oral/nasal ulcers, n(%)	25(8.4)	18(8.5)	7(8.2)	0.943
Nonscarring alopecia, n(%)	55(18.5)	36(17)	19(22.4)	0.281
Arthritis, n(%)	93(31.3)	57(26.9)	36(42.4)	0.009
Serositis, n(%)	74(24.9)	35(16.5)	39(45.9)	<0.001
Neurologic, n(%)	17(5.7)	10(4.7)	7(8.2)	0.271
Hemolytic anemia, n(%)	23(7.7)	14(6.6)	9(10.6)	0.246
Leukopenia, n(%)	155(52.2)	109(51.4)	46(54.1)	0.673
Lymphopenia, n(%)	70(23.6)	44(20.8)	26(30.6)	0.071
Thrombocytopenia, n(%)	89(30)	69(32.5)	20(23.5)	0.125
SCr, umol/L, median [IQR]	52[44–61]	52[45−60]	50[41–64]	0.432
Hematuria, n(%)	44(14.8)	20(9.4)	24(28.2)	<0.001
Pyuria, n(%)	41(13.8)	19(9)	22(25.9)	<0.001
SLEDAI-2K, median [IQR]	8[6–12]	7[5–10]	12[8.5-15]	<0.001
Hypocomplementemia, n(%)	278(93.6)	195(92)	83(97.6)	0.071
Medium-high titer aCL IgM, n(%)	13(4.9)	8(4.2)	5(6.6)	0.529
Medium-high titer aCL IgG n(%)	31(11.7)	25(13.2)	6(7.9)	0.227
Increased anti-β2GPI, n(%)	71(27.4)	56(29.9)	15(20.8)	0.141
Positive anti-dsDNA, n(%)	201(67.7)	135(63.7)	66(77.6)	0.020
Direct Coombs test, n(%)	28(15.8)	17(13.9)	11(20)	0.306
GCs use at last visit, n(%)	236(79.5)	170(80.2)	66(77.6)	0.624
HCQ use at last visit, n(%)	238(80.1)	177(83.5)	61(71.8)	0.022
ISA use at last visit, n(%)	107(36)	69(32.5)	38(44.7)	0.049
Significant proteinuria, n(%)	42(14.1)	16(7.5)	26(30.6)	<0.001
Follow-up period, months, median [IQR]	81[51-107]	85.5[57-108.75]	70[28.5-103]	0.016
Time to significant proteinuria, months, median [IQR]	46.5[20.5-86.25]	52.5[42.25-103]	37[16.5-69.25]	0.054
24-hour proteinuria(mg/d), median [IQR]	1455[899-4296.5]	1455[742.75-5995]	1610.5[1004.75-4188]	0.917

Disease duration: the time from symptom onset to the point of being newly diagnosed with SLE at our center. SCr: Serum Creatinine. Hematuria: > 5 RBCs/HPF after the exclusion of infection. Pyuria: > 5 WBCs/HPF after the exclusion of infection. SLEDAI-2K:Systemic Lupus Erythematosus Disease Activity Index 2000; Significant proteinuria: 24-hour proteinuria ≥500 mg/d; 24-hour proteinuria: the median among patients with significant proteinuria; Follow-up period: the period from diagnosis until the first occurrence of the outcome of interest (significant proteinuria) or the last follow-up, whichever occurred first; Time to significant proteinuria: the duration from the date of initial SLE diagnosis to the date of significant proteinuria occurrence,specifically for patients who developed significant proteinuria; aCL: anticardiolipin antibody; anti-β2GPI: anti-beta-2 glycoprotein I antibody; anti-dsDNA: anti-double-stranded DNA; GCs: glucocorticoids; HCQ: hydroxychloroquine; ISA: immunosuppressive agent; IQR: interquartile range.

**Fig 1:**
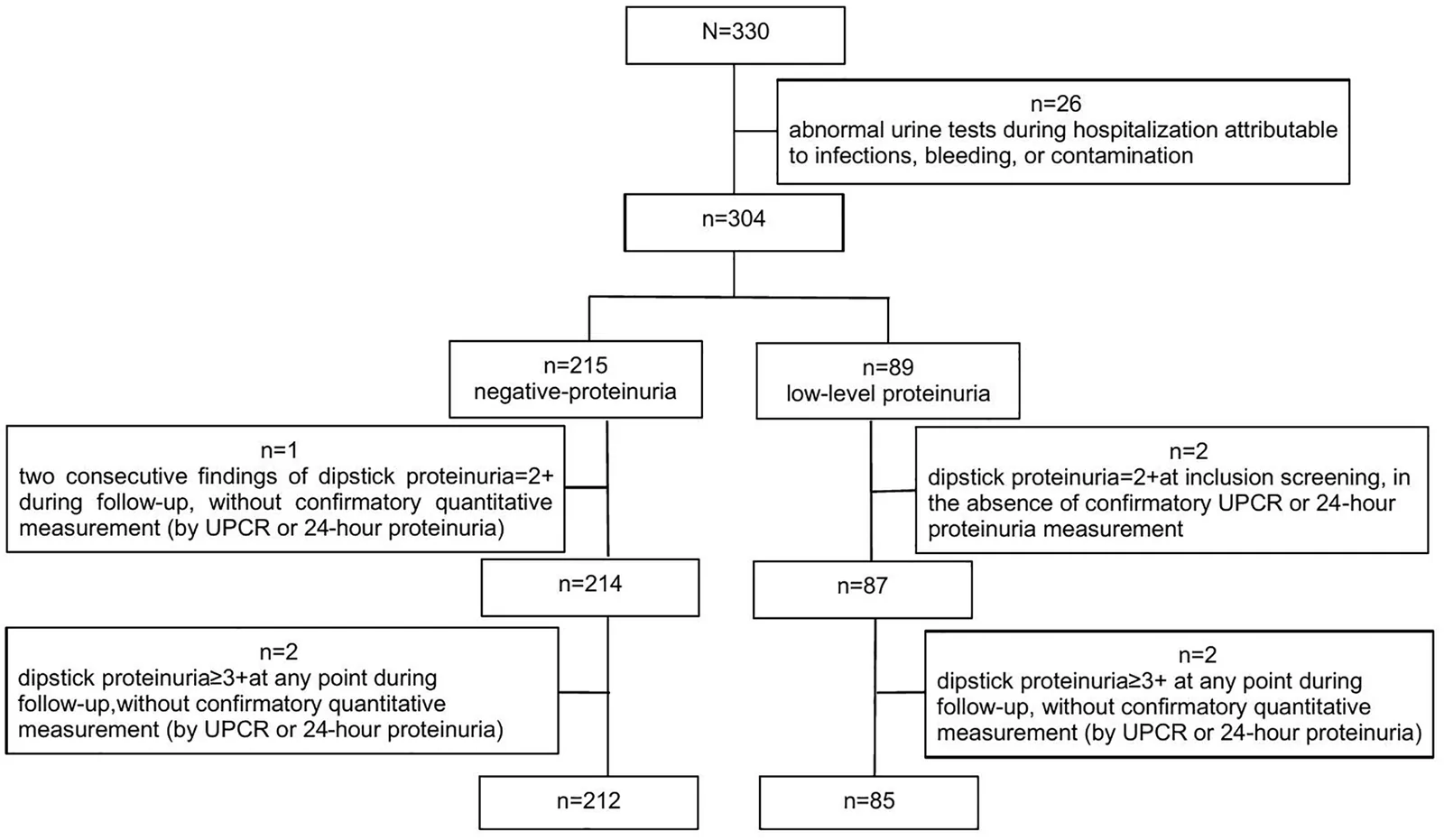
Selection and inclusion of SLE patients in the study.

### Comparison of clinical characteristics between the negative-proteinuria group and the low-level proteinuria group

There was no statistically significant difference in age between the negative and low-level proteinuria groups (median [IQR]: 33 [22–45] years vs. 30 [20.5–42] years, P > 0.05).

The low-level proteinuria group had a significantly lower proportion of female patients than the negative-proteinuria group (81.2% vs. 92%, *P* = 0.007). The median disease duration was 3 months (IQR 1–13) in the negative-proteinuria group and 2 months (IQR 1–7) in the low-level proteinuria group.The low-level proteinuria group had more frequent manifestations of fever (42.4% vs. 27.8%, *P* < 0.05), arthritis (42.4% vs. 26.9%, *P* < 0.01), and serositis (45.9% vs. 16.5%, *P* < 0.001) compared to the negative-proteinuria group, whereas no significant differences were found for other organ systems. The low-level proteinuria group had a significantly higher SLEDAI-2K score than the negative-proteinuria group (median [IQR]: 12 [8.5–15] vs. 7 [5–10]). The low-level proteinuria group exhibited significantly higher rates of hematuria (28.2% vs. 9.4%, *P* < 0.001), pyuria (25.9% vs. 9%, *P* < 0.001), and anti-dsDNA positivity (77.6% vs. 63.7%, *P* < 0.05) than the negative-proteinuria group. In contrast, the prevalence of hypocomplementemia and antiphospholipid antibody positivity was comparable between the two groups. When assessing medications being taken at the last study visit, the low-level proteinuria group had significantly fewer hydroxychloroquine (HCQ) users (71.8% vs. 83.5%; *P* < 0.05) but more immunosuppressant users (44.7% vs. 32.5%; *P* < 0.05) compared with the negative-proteinuria group, whereas the proportions of patients using corticosteroids were comparable between the two groups. The details are shown in Table 1.

### The effect of low-level proteinuria on the occurrence of significant proteinuria

Variables showing significance in bivariate analysis were included in a univariable Cox regression screening. In addition to these, patient age at diagnosis and sex were also included as covariates, based on previous studies indicating that male sex and younger age were associated with an increased risk of progressing to LN [7,8]. Univariate Cox regression analysis revealed that low-level proteinuria (*P* < 0.001, hazard ratio[HR]= 4.61, 95% confidence interval [CI]: 2.469–8.609), male sex (*P* < 0.001, HR = 3.863, 95% CI: 1.931–7.728), hematuria (*P* = 0.026, HR = 2.151, 95% CI: 1.098–4.214), and SLEDAI-2K (*P* = 0.006, HR = 1.086, 95% CI: 1.024–1.151) were associated with an increased risk of subsequent progression to significant proteinuria, whereas HCQ use conferred a protective effect against this risk(*P* = 0.004, HR = 0.404, 95% CI: 0.217–0.754). The details are shown in Fig 2. After adjusting for confounders in the multivariable model, low-level proteinuria significantly increased the risk of significant proteinuria (P < 0.001, HR = 3.355, 95% CI: 1.6646.765), as did male sex (*P* = 0.001, HR = 3.178, 95% CI: 1.563–6.460) (Fig 3 A). The findings remained unchanged in the sensitivity analysis addressing insufficient follow-up (Fig 3 B). Kaplan-Meier estimates of significant proteinuria -free survival stratified by the presence of low-level proteinuria at the initial diagnosis of SLE are shown in Fig 4.

**Fig 2:**
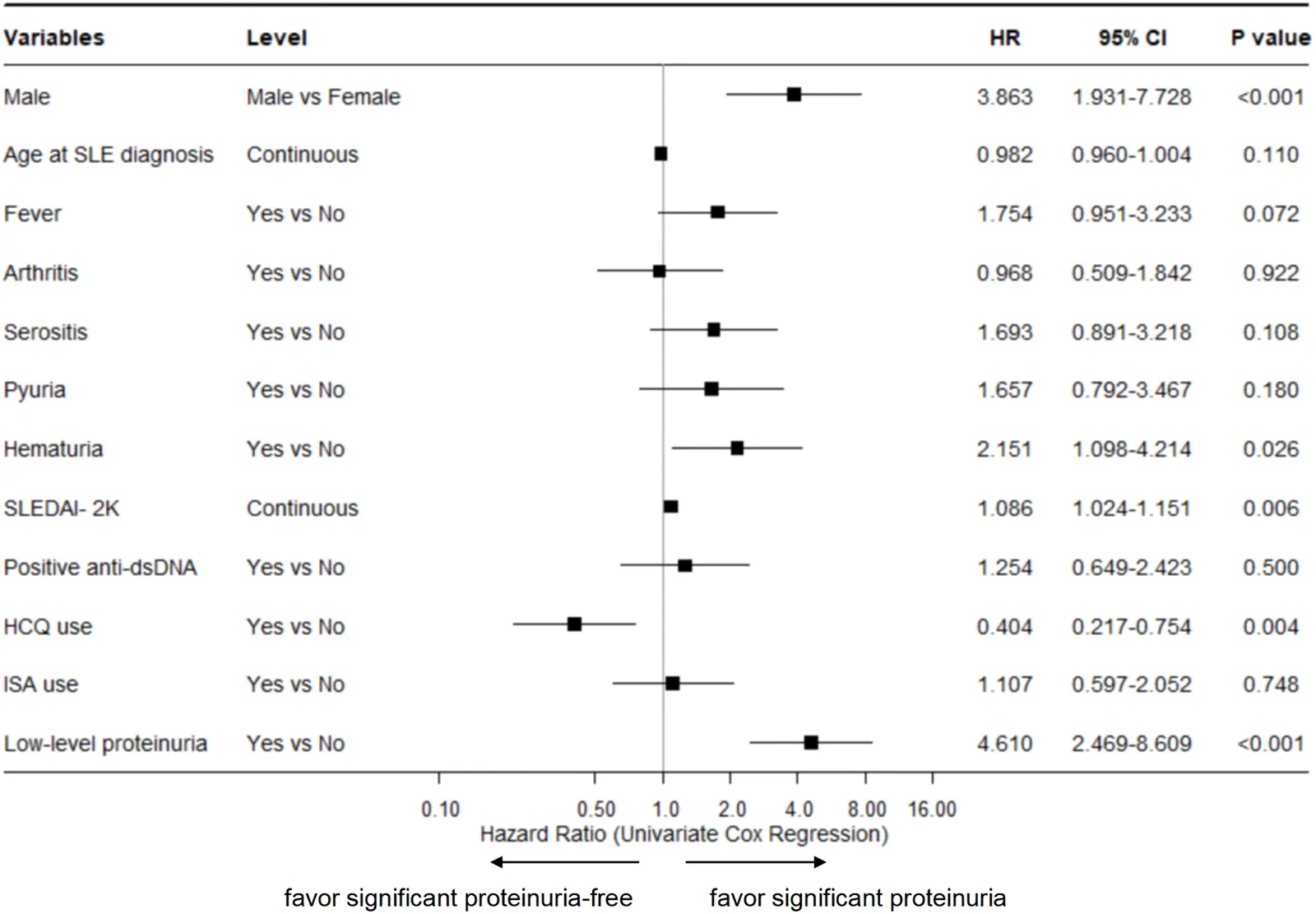
Baseline predictors for significant proteinuria according to univariate Cox regression analysis. The figure presents the HRs and 95% CIs associated with the outcome. HR: hazard ratio. See Table 1 note for detailed explanations of other terms.

**Fig 3:**
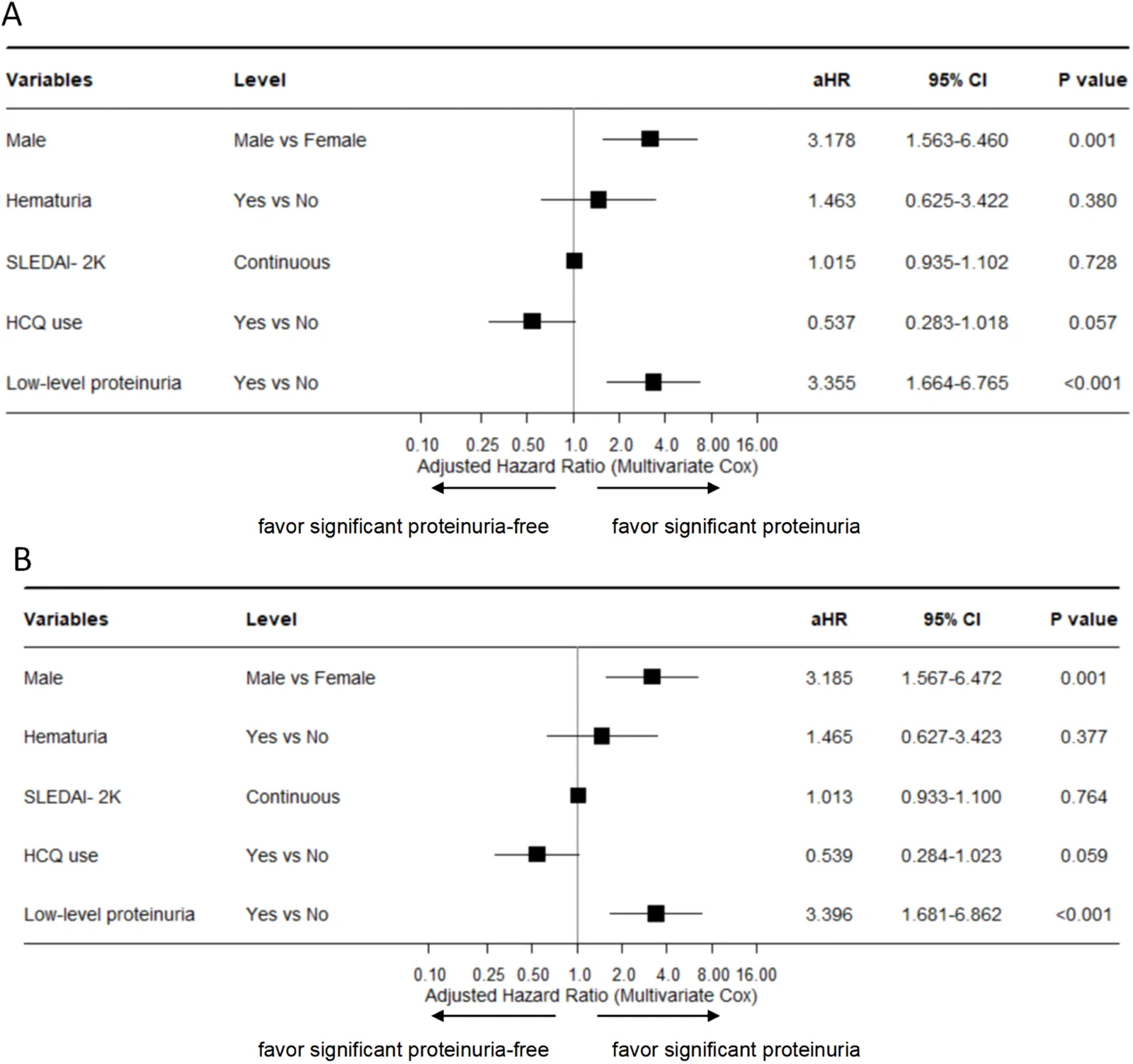
Multivariate Cox regression analysis of the effect of low-level proteinuria on the occurrence of significant proteinuria. **(A)** Primary analysis in the full cohort; **(B)** Sensitivity analysis excluding patients in the bottom quartile of follow-up duration (<20.5 months) without the outcome, to assess robustness. The HRs and 95% CIs are associated with the outcome. HR: hazard ratio. See Table 1 note for detailed explanations of other terms.

**Fig 4:**
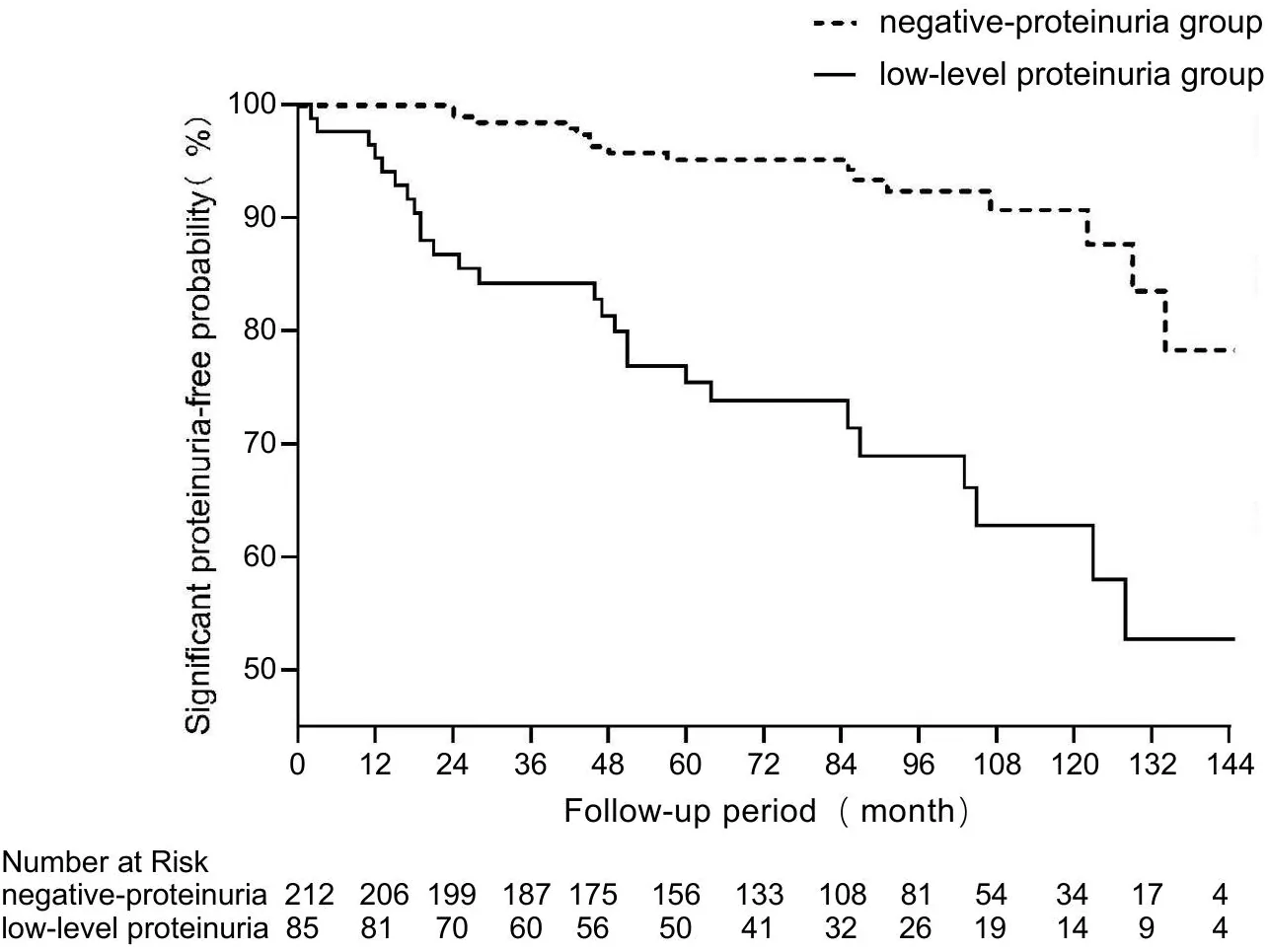
Kaplan-Meier estimates of significant proteinuria-free survival stratified by the presence of low-level proteinuria at the initial diagnosis of SLE.

## Discussion

Our study assessed the association between low-level proteinuria present at the initial diagnosis of SLE and the subsequent risk of developing overt LN—characterized by significant proteinuria. To our knowledge, this is the first study to show that the presence of low-level proteinuria at the initial diagnosis of SLE significantly elevated the risk for subsequent development of overt LN, defined by significant proteinuria. This presence elevated the progression risk by 235.5%.

LN complicates the course of 25–60% of systemic lupus erythematosus (SLE) cases, with its incidence and prevalence influenced by demographics and socioeconomic status [9]. It is not uncommon for SLE to be diagnosed concurrently with lupus nephritis (LN) at the initial presentation. Our inception lupus cohort demonstrated that 21.72% of patients had lupus nephritis (LN) at the time of initial SLE diagnosis [10]. Additionally, a proportion of patients who were initially LN-negative progressed to LN during follow-up. As shown in Table 1, among the 297 SLE patients with non-significant proteinuria at the initial diagnosis of SLE, 42 (14.1%) progressed to LN characterized by a significant increase in proteinuria. Despite an increasing understanding of disease biology [11] and more effective treatment options, such as new immunosuppressive agents [12], biologics like belimumab [13], and B-cell-depleting agents including rituximab [14], LN remains a substantial cause of morbidity and mortality as it can lead to irreversible kidney failure and associated complications. To date, LN represents the most severe organ manifestation of SLE in terms of morbidity and mortality.

Thus, with currently available treatments, early recognition of individuals at high risk for LN and delivering suitable intervention is a pivotal measure for improving its prognosis. Multiple predictors for the development of LN after diagnosis of SLE have been identified in previous studies. In summary, male sex and younger age at disease onset were well-recognized potential predictors for LN [7,8,15]. Similarly, our study also found that male patients had a 217.8% higher risk of developing significant proteinuria during the disease course than females (HR = 3.178, 95% CI: 1.563–6.460,Fig 3A).

Currently, 0.5 g of 24-hour urine protein is widely adopted as a conventional cutoff and one of the *classification* criteria for LN [6,16]. However, some patients present with sub-0.5g 24-hour urine protein at the initial diagnosis of SLE. Whether this early low-level proteinuria signifies any clinical relevance, particularly as a predictor for progressing to clinically defined LN, remains uninvestigated. In our study, patients with low-level proteinuria at initial diagnosis had a significantly higher incidence of subsequent LN compared to those without it (30.6% vs. 7.5%, *P* < 0.001; Table 1). Due to baseline differences between patients with and without low-level proteinuria, we first performed univariable Cox regression to determine whether low-level proteinuria was associated with an increased risk of subsequent LN. The analysis identified low-level proteinuria, male sex, hematuria, and high SLEDAI-2K score as baseline predictors for LN progression, whereas HCQ use was associated with a reduced risk.

Furthermore, to control for potential confounders, we performed a multivariable Cox regression analysis. The result demonstrated that low-level proteinuria remained an independent predictor factor for subsequent LN (HR 3.355, 95% CI: 1.664–6.765; Fig 3 A). The Kaplan-Meier curve clearly demonstrates the significant impact of low-level proteinuria on the subsequent development of LN (Fig 4)。The findings from the sensitivity analysis, which excluded patients with follow-up <20.5 months (n = 14), were consistent with the primary analysis. The association between low-level proteinuria and LN progression remained significant (HR 4.626, 95% CI: 2.477–8.638), confirming the robustness of our primary results.

Current guidelines recommend corticosteroids and immunosuppressants for patients with major organ involvement, such as LN. However, for those without such involvement, initiating immunosuppressive therapy is not routinely recommended [1]. As a fundamental baseline treatment for SLE, HCQ has been universally endorsed by guidelines for years, reflecting a long-standing consensus on its importance in reducing disease activity and preventing disease flare [17–19]. HCQ maintenance can exert a protective role in preventing disease relapse after GCs withdrawal [20]. Our study also suggested a trend that HCQ use was associated with a reduced risk of progressing to LN among SLE patients. For SLE patients with low-level proteinuria who are at high risk for subsequent LN, it remains unestablished whether treatment with HCQ, with or without corticosteroids, constitutes sufficient management. Conversely, whether initiating an additional immunosuppressant at diagnosis yields a more favorable risk-benefit profile for preventing subsequent LN is unknown. The potential benefit of early immunosuppressive therapy in this specific patient population warrants further investigation.

Our study has several limitations. Firstly, our patient population was derived from a single center(Fujian Medical University Union Hospital) with a relatively small sample size, which may limit the generalizability of our findings. Validation in larger, multi-center cohorts is required. Additionally, due to the retrospective design, it was not feasible to perform 24-hour urine protein quantification at diagnosis for all SLE patients, irrespective of their routine urinalysis results. This could introduce a potential selection bias. Therefore, high-quality prospective cohort studies are warranted to confirm our results.

In conclusion, this is the first study to suggest that the presence of low-level proteinuria at the initial diagnosis of SLE significantly increases the risk of subsequently developing LN, which is characterized by significant proteinuria. This study provides crucial data that can assist clinicians in identifying SLE patients at elevated risk for future LN. This knowledge enables more vigilant monitoring and the formulation of personalized management strategies in routine practice. To definitively address the risk and optimal intervention for patients with low-level proteinuria at initial diagnosis, a large-scale, multi-center, prospective interventional cohort study is warranted.
